# Open-source real-time quantitative RT-PCR-based on a RNA standard for the assessment of SARS-CoV-2 viral load

**DOI:** 10.1590/0074-02760210237

**Published:** 2022-01-28

**Authors:** Juliana Comerlato, Carolina Baldisserotto Comerlato, Fernando Hayashi Sant’Anna, Marina Bessel, Celina Monteiro Abreu, Eliana Márcia Wendland

**Affiliations:** 1 Hospital Moinhos de Vento, Porto Alegre, RS, Brasil; 2 Johns Hopkins School of Medicine, Department of Molecular and Comparative Pathobiology, Baltimore, MD, United States; 3 Universidade Federal de Ciências da Saúde de Porto Alegre, Departamento de Saúde Pública, Porto Alegre, RS, Brasil

**Keywords:** reverse transcriptase polymerase chain reaction, real-time polymerase chain reaction, SARS-CoV-2, COVID-19, viral load, COVID-19 testing

## Abstract

**BACKGROUND:**

Detection of severe acute respiratory syndrome coronavirus 2 (SARS-CoV-2) target genes by molecular methods has been chosen as the main approach to identify individuals with Coronavirus disease 2019 (COVID-19) infection.

**OBJECTIVES:**

In this study, we developed an open-source RNA standard-based real-time quantitative RT-PCR (RT-qPCR) assay for quantitative diagnostics of SARS-CoV-2 from nasopharynx, oropharynx, saliva and plasma samples.

**METHODS AND FINDINGS:**

We evaluated three SARS-CoV-2 target genes and selected the RNA-dependent RNA polymerase (RdRp) gene, given its better performance. To improve the efficiency of the assay, a primer gradient containing 25 primers forward and reverse concentration combinations was performed. The forward and reverse primer pairs with 400 nM and 500 nM concentrations, respectively, showed the highest sensitivity. The LOD_95%_ was ~60 copies per reaction. From the four biological matrices tested, none of them interfered with the viral load measurement. Comparison with the Allplex^TM^ 2019-nCoV assay (Seegene) demonstrated that our test presents 90% sensitivity and 100% specificity.

**MAIN CONCLUSIONS:**

We developed an efficient molecular method able to measure absolute SARS-CoV-2 viral load with high replicability, sensitivity and specificity in different clinical samples.

Diagnostic molecular assays have been playing an essential role in the pandemic scenario of severe acute respiratory syndrome coronavirus 2 (SARS-CoV-2), monitoring the suspected and confirmed Coronavirus disease 2019 (COVID-19) cases and the dynamics of virus infection. Given this, these analyses are invaluable resources to guide decision-making of the public healthcare system. The gold standard for SARS-CoV-2 diagnosis is real-time reverse transcription polymerase chain reaction (RT-PCR), which is a sensitive method that can rapidly confirm an infection a few days after exposure.^1^ However, the method is often qualitative (i.e., only providing informing about the presence/absence of the virus but not its quantity). The evaluation of the viral load could aid the understanding of the course of the pathogenic process and therefore could provide information for the management of the infection.^2^

In addition to the conventional qualitative assay, real time quantitative reverse transcription PCR (RT-qPCR) has the advantage of assessing the viral load by means of absolute quantification, using as reference a standard curve of a known quantity of the target nucleic acid. Concerning the seriousness of the pandemic crisis, open-source, affordable and reproducible methods for absolute quantification of viral load should be promptly available, avoiding unnecessary costs for research projects aiming to unveil the viral cycle and to develop novel therapies.

In this sense, we developed a sensitive and specific absolute quantification RNA-dependent RNA polymerase (RdRp) RT-PCR assay, based on RNA transcript standards, and validated on nasopharyngeal (NP), oropharyngeal (OP), saliva and plasma biological samples.

## MATERIALS AND METHODS

*Study approval* - The Research Ethics Committee of Hospital Moinhos de Vento approved this study under the protocol number 3977144. All participants provided a written informed consent, and the study was conducted according to Good Laboratory Practices and the Declaration of Helsinki.

*Biological samples* - The study included 86 samples from 47 individuals who previously tested positive for SARS-CoV-2 in a qualitative RT-PCR assay between 24 July 2020 and 4 September 2020 at Hospital Moinhos de Vento (Porto Alegre, RS - Brazil). We then collected NP (both nostrils) and/or OP swab samples from those individuals who presented with different days of symptoms onset at the time of sampling, within 48 h after the first diagnosis [Supplementary data (Table I)]. For the specimen background interference assay, we collected NP, OP, saliva and blood samples from a group of eight participants without clinical suspicion of COVID-19 and negative RT-PCR for SARS-CoV-2.

NP and OP sampling were performed according to the CDC recommendations^3^ using FLOQSwab^®^ with eNAT medium (COPAN Diagnostics Inc.). Saliva collection (1 mL) was performed by self-collection in a sterile flask. Thereafter, 2 mL of eNAT medium was added to the sample. Peripheral blood samples were collected in 5 mL EDTA tubes (BD). Samples were stored at 4ºC and processed within 3days after their collection. NP and OP samples were homogenized by vortex for 15 seconds. EDTA blood tubes were centrifuged at 400 g for 15 minutes to separate the plasma, and the saliva samples were directly aliquoted. All samples were stored at -80ºC until RNA extraction.

*Nucleic acid extraction* - The nucleic acid extraction was carried out using 300 µL of the clinical sample with the Maxwell^®^ RSC Viral Total Nucleic Acid Purification Kit and the Maxwell^®^ Instrument, according to the manufacturer’s instructions. Total nucleic acids were eluted in 50 µL of water.

*Primer and probes designs* - SARS-CoV-2 sequences available in the GenBank on 7 May 2020 were aligned using the IDT PrimerQuest^TM^ tool.^4^ Primers and probes were designed using conserved regions of nucleoprotein (N), RdRp and spike glycoprotein (S) of SARS-CoV-2 sequences as references. The following parameters were considered: a length of 18-30 nucleotides for primers; melting temperature (Tm) around 65ºC within a maximum of 5ºC difference between the primer pair; and the GC content was defined between 40% and 60%, with the 3’ of a primer ending in C or G to promote binding. We checked for hairpins, self-dimers and heterodimers using the online IDT oligoanalyzer tool.^5^ The oligonucleotides generated were submitted to nucleotide BLAST search on the NCBI (blastn) web server to ensure specificity of the sequences.

*Synthesis of standard RNA templates* - The target sequences of N, RdRp and S genes were reverse transcribed (High Capacity RNA Kit, ThermoFisher Scientific) and amplified (Platinum Taq DNA Polymerase High Fidelity Kit, ThermoFisher Scientific) using the primers described in Table I and a local SARS-CoV-2-positive sample. The amplicons were cloned in the expression vector pCR™2.1^®^ and then transformed to MAX efficiency^TM^ Stbl2^TM^ cells (ThermoFisher Scientific). Transformants were plated onto solid Luria Bertani (LB) medium containing 100 µg/mL ampicillin and incubated overnight at 37ºC. Colonies were then screened by gene-specific PCR.

**TABLE I:** Primer-probe sets sequence of the nucleoprotein (N), RNA-dependent RNA polymerase (RdRp) and spike glycoprotein (S) genes of severe acute respiratory syndrome coronavirus 2 (SARS-CoV-2)

Target	Primer/Probe	Sequence
N	SARS-CoV-2 GENE N_PF	5’ CTCAGTCCAAGATGGTATTTC 3’
	SARS-CoV-2 GENE N_PR	5’ TAGCACCATAGGGAAGTC 3’
	SARS-CoV-2 GENE N_Pr	5’ FAM-TAGGAACTGGGCCAGAAGCT-BHQ1 3’
RdRp	SARS-CoV-2 GENE RdRp JC_PF	5’ AGGTAGTGGAGTTCCTGTTG 3’
	SARS-CoV-2 GENE RdRp JC_PR	5’ GTCAACATGTGACTCTGCAG 3’
	SARS-CoV-2 GENE RdRp JC_Pr	5’ FAM-GTTAATGCCTATATTAACCTTGACCAGGGC-BHQ1 3’
S	SARS-CoV-2 GENE S_PF	5’ TATACATGTCTCTGGGACCAATG 3’
	SARS-CoV-2 GENE S_PR	5’ ATCCAGCCTCTTATTATGTTAGAC 3’
	SARS-CoV-2 GENE S_Pr	5’ FAM-ACTAAGAGGTTTGATAACCCTGTCCTACC-BHQ1 3’

Plasmids containing the inserts were extracted using the QIAprep Spin Miniprep Kit (QIAGEN™) and further submitted to Sanger sequencing. The sequencing reaction was performed following the protocol of the BigDye^®^ Terminator Sequencing Kit (ThermoFisher Scientific) in the 3100 ABI PRISM automatic sequencer (Applied Biosystems, USA). Raw reads were processed and assembled using the Staden package program.^6^ To check the integrity of insert sequences, we compared them to the Wuhan-Hu-1 genome sequence (GenBank accession number: NC_045512.2) with blastn tool.

Each of the RNA templates (N, RdRp and S) were transcribed *in vitro* using the Megascript T7 Transcription Kit (Ambion by Life Technologies) as described by the manufacturer and later purified using the TURBO DNA-Free™ Kit (ThermoFisher Scientific). RNA transcripts were quantified using the Qubit™ RNA HS Assay Kit (ThermoFisher Scientific). In order to verify the uniqueness of each RNA template, the transcripts were denatured at 65ºC for 5 minutes and immediately cooled on ice. Subsequently, they were migrated in conventional SYBR Green 2% agarose gel.

*Analysis of PCR efficiency of the N, RdRp and S transcripts* - To generate qPCR standard curves for absolute quantification, serial 10-fold dilutions of known quantities of N, RdRp and S RNA transcripts were performed. Initially, the RT-qPCR assays for each gene were carried out with the same reagents and conditions. For this purpose, we performed the RT-qPCR using the SuperScript^TM^ III Platinum^TM^ One-Step qRT-PCR system (Invitrogen, USA), with 5 µL of the standard RNA transcript dilution (10^2^ to 10^9^ copies per reaction), 200 nM of each primer and 100 nM of probe in a final volume of 25 µL. The standard cycling programme was set with the default parameters of the manufacturer. The primer-probe sets are described in Table I. The threshold was automatically calculated to 0.04 for all genes. However, the threshold bar of the RdRp RT-qPCR assay had to be set to 0.02 to position it in the exponential phase of the curve. The result of the RT-qPCR assay was considered positive for SARS-CoV-2 when the cycle threshold (Ct) was < 40.

*Primer gradient and limit of detection* - To improve the efficiency and sensitivity of the RdRp RT-qPCR assay, we optimized the primer concentration. A matrix of different concentrations of each primer (100 nM to 500 nM), was built, totalling 25 combinations. Each reaction condition was carried out in duplicate with 10^3^ copies of RdRp RNA transcripts. For determination of the 95% limit of detection (LOD_95%_), we evaluated 20 measurements of each standard RNA in the concentrations of 1, 10 and 100 copies per reaction.

*Background interference using the biological matrix of clinical samples* - We evaluated the interference of the NP, OP, saliva and plasma biological background in the RdRp RT-qPCR assay. For this purpose, eight negative samples of each specimen were pooled, and 5 µL of the pool was included in the reaction mix of the RdRp RT-qPCR assay. For each specimen background, a standard curve was run and compared to a non-spiked standard curve (only water).

*Sensitivity and specificity of the RdRp RT-qPCR assay* - To assess the sensitivity and specificity of the in-house RdRp RT-qPCR assay, we concurrently compared it with the Allplex™ 2019-nCoV assay, which is a commercial test that detects the RdRp, N and E genes of SARS-CoV-2, using the samples listed in the Supplementary data (Table I). We utilized 5 µL of viral RNA for each PCR volume reaction of 25 µL. The Ct results from the Allplex assay were compared with the Ct and the copy number results from the RdRp RT-qPCR assay. The results of each sample from both methodologies were compared to establish a linear regression in order to predict the amount of RNA from the Allplex Ct results.

*Reproducibility and analytical specificity analyses* - The repeatability of the technique was determined by comparing standard curves from nine independent assays. The coefficient of variation (CV) was determined using the formula: CV = (SD [Ct value]/general average [Ct value]) × 100. For analytical specificity analysis, 4026 sequences of the Nextstrain global dataset (18 May 2021) were downloaded from the GISAID database. Nextclade CLI was utilized for determining the nucleotide substitutions of the global sample. The relative frequency of sequences presenting substitutions along the RdRp gene of the Wuhan-Hu-1 genome was plotted using a script written in Python.

*Statistical analysis* - A linear regression model was used to explain the relationship between the RdRp RT-qPCR assay and Allplex™ 2019-nCoV assay. R^2^ was used to determine the percentage of the RdRp RT-qPCR assay variation explained by the Allplex™ 2019-nCoV assay. Specificity and sensitivity with corresponding 95% confidence intervals (CIs) were calculated to identify the positive and negative results for the RdRp RT-qPCR assay in accordance with the results of the RT-PCR Allplex™ 2019-nCoV assay.

## RESULTS

**Fig. 1:**
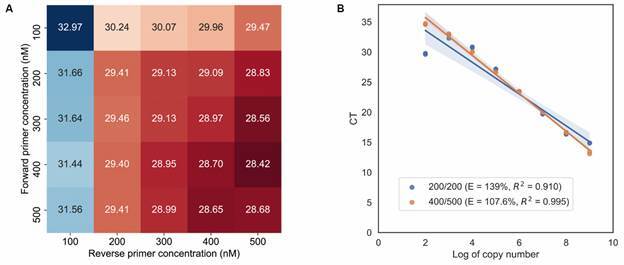
RNA-dependent RNA polymerase (RdRp) real-time quantitative polymerase chain reaction (RT-qPCR) optimization using a primer concentration matrix approach. Heatmap showing the Cycle threshold (Ct) values according to the primer concentrations. Forward primer concentrations are in the rows. Reverse primer concentrations are in the columns. Cts values are represented by colour (blue, higher Ct; red, lower Ct). The lowest Ct value was found for the combination of 400 nM forward primer and 500 nM reverse primer (A); Efficiency (E) and coefficients of determination (*R* ^*2*^ ) of the RdRp RT-qPCR standard quantification curves using primer F and R concentration of 400/500 nM versus 200/200 nM. Slope: -3.15, iIntercept: 42.04, data from the 400/500 nM concentration line (B).

*Standardization of an in-house RT-qPCR assay for SARS-CoV-2* - Different parameters were evaluated during the development of the RT-qPCR assay for the SARS-CoV-2 N, RdRp and S genes. Among the three plasmid constructs, the one containing the RdRp region presented the highest minipreparation yield (data not shown), while that containing the S region presented the lowest yield. The plasmids were then utilized as templates for in vitro RNA synthesis. The RNA products of each gene fragment were presented as single bands on the agarose gel after the denaturation process [Supplementary data (Fig. 1)].

**Fig. 2:**
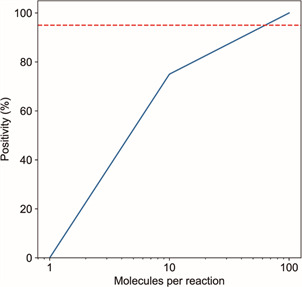
determination of the quantitative polymerase chain reaction (qPCR) 95% limit of detection (LOD_95%_). The positivity rates of reactions containing 1, 10 or 100 molecules of the RNA-dependent RNA polymerase (RdRp) transcript (20 replicates for each quantity). PCR reactions presenting Cycle threshold (Ct) values below 40 are considered positive. Traced line shows the minimum positivity rate for determining the detection limit.

In the preliminary RT-qPCR assay using default parameters, the RdRp gene presented the highest efficiency, although suboptimal [Supplementary data (Fig. 2)]. Therefore, we decided to improve the protocol of the in-house RT-qPCR assay for the target RdRp gene.

*Optimization and LOD*_*95%*_ *of the RdRp RT-qPCR assay* - In the primer concentration matrix assay for the RdRp gene fragment, we verified that the lowest Ct mean was obtained when using 400 nM of the forward primer and 500 nM of the reverse primer (Fig. 1A). We observed that when using the 400/500 nM primer combination, the efficiency of the in-house RT-qPCR technique was 107.6%, while the efficiency of the curve using both primers at the concentration of 200 nM was 139% (Fig. 1B).

In the LOD_95%_ assay, 75% of the reactions containing 10 RNA copies were positive with a Ct mean of 36.74 (SD of 2.38). On the other hand, all reactions containing 100 RNA copies were positive. We estimated that the LOD_95%_ was approximately ~60 copies per reaction, considering where the line crosses the threshold of 95% positivity (Fig. 2).

**Fig. 3:**
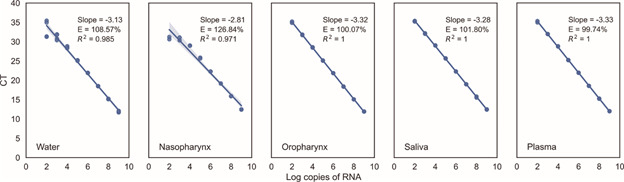
RNA copy number variation using water or different biological matrices in the RNA-dependent RNA polymerase (RdRp) real-time quantitative polymerase chain reaction (RT-qPCR) assay. The variation in viral load using RNA from clinical samples of the upper respiratory tract, saliva and plasma were compared with the results of the control using water.

*Interference assay of biological matrices in viral load measurements* - The use of four different biological matrices through eight clinical samples negative for SARS-CoV-2 did not affect the RT-qPCR assay efficiency between the interval of 10^3^ to 10^9^ copies of RNA per reaction (Fig. 3).

**Fig. 4:**
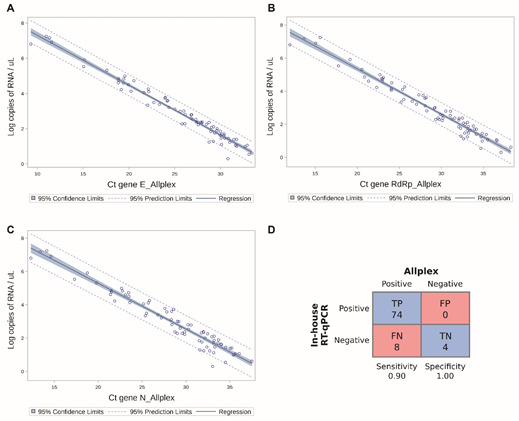
concordance of the RNA-dependent RNA polymerase (RdRp) real-time quantitative polymerase chain reaction (RT-qPCR) assay with the commercial RT-PCR Allplex™ 2019-nCoV Assay. RdRp RT-qPCR assay versus the gene N of the Allplex assay (A); In-house RdRp RT-qPCR assay versus the gene E of the Allplex assay (B); RdRp RT-qPCR assay versus the gene RdRp gene of the Allplex assay (C). The linear regression model showed that the Ct results from the N, E and RdRp genes obtained from the Allplex assay were comparable with the RNA copies detected by the RdRp RT-qPCR as to nasopharynx and oropharynx samples. The R^2^ of the RdRp, N and E genes was 0.95, 0.94 and 0.96 with 95% of confidence intervals limits. (D) Confusion matrix depicting the RdRp RT-qPCR assay results in relation to those of the Allplex assay. Sensitivity and specificity were calculated from the values shown in the confusion matrix. Ct: Cycle threshold; TP: true positive; TN: true negative; FP: false positive; FN: false negative.

*Concordance of the clinical performance of the RdRp RT-qPCR assay with the commercial RT-PCR Allplex™ 2019-nCoV assay* - Although, at first, the samples were obtained from individuals who previously tested positive for SARS-CoV-2, four samples were found to be negative in our RdRp RT-qPCR assay and in the Allplex™ 2019-nCoV assay, then being considered true negatives [Supplementary data (Table I)]. In general, our RdRp RT-qPCR assay presented a linear correlation to the three genes of the Allplex™ 2019-nCoV Assay (Fig. 4); nevertheless, eight samples were false negatives. Remarkably, the average Ct values of all false negative samples in the Allplex assay were 34.13 (gene E), 36.81 (gene RdRp) and 36.96 (gene N), while those from the true positive samples were 25.4 (gene E), 28.78 (gene RdRp) and 28.49 (gene N).

Four of the false negative samples did not present detectable amplification of the RdRp gene in the Allplex assay, but they showed amplification for at least the N gene [Supplementary data (Table I)]. Considering the total of discordant results, the RdRp RTq-PCR assay showed a sensitivity of 0.90 and specificity of 1.00 (Fig. 4D).

*Assay reproducibility* - In further qPCR experiments, we analysed multiple standard curves and demonstrated that the interassay variability was lower than 7% [Table II; Supplementary data (Table II)].

**TABLE II:** Reproducibility assay. The reproducibility assay was performed in nine runs on different days showing the calculation of the coefficient of variation (CV%) between runs

Quantity^*^	Ct mean	Ct SD	CV (%)
100	34.1848	2.077024	6.075871
1000	32.24935	1.465892	4.545493
10000	29.00559	0.862914	2.974991
100000	25.55972	0.636771	2.491307
1000000	22.43004	0.678741	3.026036
10000000	18.93573	0.560612	2.960605
100000000	15.45206	0.644352	4.170007
1000000000	12.08888	0.689905	5.706937

*: number of RNA-dependent RNA polymerase (RdRp) transcripts copies per reaction tube. SD: standard deviation; CV: coefficient of variation.

**Fig. 5:**
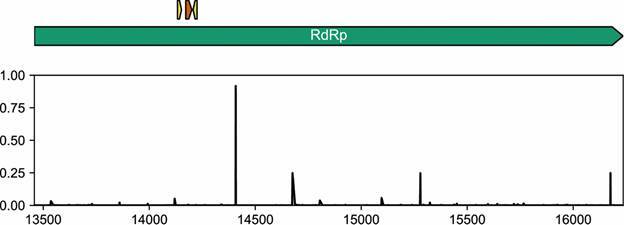
analytical specificity of the RNA-dependent RNA polymerase (RdRp) real-time quantitative polymerase chain reaction (RT-qPCR) assay. The green arrow represents the RdRp gene, the small yellow arrows represent the primer binding sites and the small orange arrow represents the probe binding site. The barplot shows the relative frequency of sequences presenting mutations per site of the RdRp gene from samples found worldwide. Few sequences present substitutions in the primers and probe sites with respect to the Wuhan-Hu-1/2019 sequence.

*Analytical specificity* - As long as the pandemic is ongoing, the number of viral genome sequences are accumulating in the GISAID database. Given this, we evaluated the proportion of sequences of a global subsample that presents mutations in the RdRp target region. From more than 4000 sequences evaluated, the number of sequences presenting substitutions per position were lower than 0.6% in the considered target region (Fig. 5).

## DISCUSSION

In-house RT-qPCR assays have been developed and evaluated, particularly in developing countries, because affordable and generalized access to the SARS-CoV-2 RNA viral load is urgently needed in the context of expected universal access for the prevention and study of novel drugs for its treatment. We describe the development of a qPCR methodology for the molecular quantification of a novel in-house RT-qPCR assay for different biological specimens from individuals infected with SARS-CoV-2.

Our assay is a quantitative RT-PCR that utilizes a standard curve as a reference for quantifying the viral load in the samples. In contrast, many studies have been using Ct values of qualitative diagnostic tests as a proxy of viral load.^7^ ^,^ ^8^ ^,^ ^9^ However, this practice is not advised, since it does not consider the correspondence limits between the Ct and viral load.^10^ Furthermore, the inter-run heterogeneity and inconsistency can be additional hurdles to achieve precise quantification data based only on Ct values.^11^

Considering the chemical nature of the genetic material of SARS-CoV-2, we set up a standard curve using defined amounts of RNA representing a portion of the RdRp gene. In this sense, we aimed to better emulate the real conditions of reverse transcription and amplification that the genomes of coronaviruses undergo in the process of molecular detection. On the other hand, a common procedure of quantitative assays is using DNA templates as references.^12^ ^,^ ^13^ However, this strategy underestimates the total number of viral RNA molecules in samples, since the underlying assumption is that reverse transcription is 100% efficient, which may not always be the case.^14^

Comparing our technique to the Allplex™ 2019-nCoV Assay, which is a clinical test used worldwide, we observed eight false negatives (negative in our assay and positive in the Allplex assay). Further investigation indicated that these discordant samples presented high Ct values in the Allplex assay independently of the onset of symptoms. Although all of these samples presented amplification of the gene N, four of them did not show amplification of the RdRp target in the Allplex assay. Since the PCR template input is composed of viral genomic RNA and subgenomic mRNAs, the heterogeneous amplification of each gene may be due to their differential RNA stability and/or transcription.^15^ Nevertheless, coincidentally, the RdRp-SARSr primer-probe set designed by the University Hospital Charité also presented a lower sensitivity than assays based on other viral gene targets, but it was given a mismatch between the reverse primer and circulating SARS-CoV-2.^16^ However, we verified, by an in silico analysis, that our primer-probe set for the RdRp gene is suitable for maintaining the assay’s analytical specificity due to the sequence conservation of the target region.

Our methodology also proved to be efficient under biological matrices other than NP samples, which is the gold standard specimen for the diagnosis of SARS-CoV-2.^11^ ^,^ ^17^ ^,^ ^18^ Other studies demonstrated the importance of using different specimens for evaluating the SARS-CoV-2 viral load to understand the dynamics of the viral infection.^19^ ^,^ ^20^

Considering that the time since the onset of symptoms is an important confounder in the interpretation of the causes and consequences of a high SARS-CoV-2 viral load^21^ we cannot predict severity and/or disease progression only based on viral quantification. In fact, there has been some controversy regarding the use of the SARS-CoV-2 viral load as a prognostic indicator of disease severity._^22^_ ^,^ ^23^ Therefore, the application of viral load assessment in the clinical setting is for now limited. On the other hand, we acknowledge diverse applications of the SARS-CoV-2 viral load assessment for research purposes.

Viral load quantification can be used for filling knowledge gaps regarding the pathophysiology of the virus, disease progression and treatment and prevention of nosocomial infections._^24^_ ^,^ ^25^ Currently, multiple efforts are evaluating the effects of chemotherapy and vaccination on the SARS-CoV-2 cycle, which are useful for developing novel therapies and prevention measures._^26^_ ^,^ ^27^ ^,^ ^28^ ^,^ _^29^_ Additionally, the SARS-CoV-2 viral load has been investigated for surveillance of wastewater contamination, allowing the early prediction of pandemic waves and the indirect monitoring of the number of COVID-19 cases in a population of interest.^30^ ^,^ ^31^ ^,^ ^32^

One of the main advantages of our assay is that it is open-source, which enables its adaptation to different laboratory settings, besides allowing future improvements of the protocol. In this sense, our flexible assay could be adjusted for COVID-19 qualitative diagnosis without the need of using a standard curve for quantification.

*In conclusion* - The novel assay reported here, RdRp RT-qPCR, stands out for its robustness, high efficiency and sensitivity, providing results in a relatively short time frame. Our study developed a tool that can generate valuable data for understanding COVID-19, with a significantly lower running cost compared to commercially available assays. The sensitive RdRp RT-qPCR assay could help to overcome the cost barriers and serve as a useful addition to the currently limited SARS-CoV-2 viral load assay options for monitoring the infection in resource-limited settings.
