# Supplementary material for: Open-source real-time quantitative RT-PCR-based on a RNA standard for the assessment of SARS-CoV-2 viral load
Source: Mem Inst Oswaldo Cruz. 2022 Jan 28;116:e210237. doi: 10.1590/0074-02760210237 (PMC8803346; doi:10.1590/0074-02760210237)
Supplement: Supplementary file 1 [file 1678-8060-mioc-116-e210237-s.pdf]

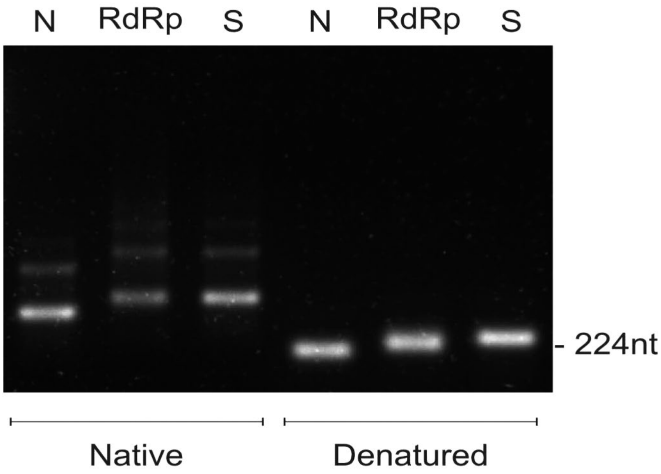

Fig. 1: purity of the RNA templates used in the optimization of the real time quantitative reverse transcription PCR (RT-qPCR) assay. Transcripts from the nucleoprotein (N), RNA-dependent RNA polymerase (RdRp) and spike glycoprotein (S) target sequences from severe acute respiratory syndrome coronavirus 2 (SARS-CoV-2) were denatured by heating, followed by cooling and migrated in agarose gel electrophoresis. The native samples, without a denaturation protocol, of each RNA were migrated in parallel to compare the pattern of migration. The denatured N, RdRp and S transcripts have 196 nt, 224 nt and 244 nt, respectively.

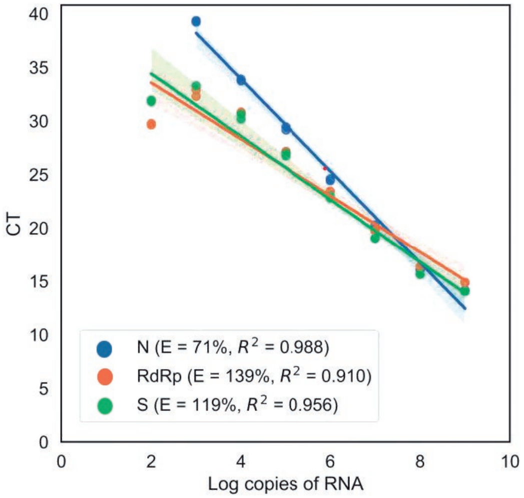

Fig. 2: standard quantification curves of the nucleoprotein (N), RNA-dependent RNA polymerase (RdRp) and spike glycoprotein (S) genes. Linear regression analysis of 10 serial dilutions using the RNA transcripts of the N, RdRp and S genes from a nasopharyngeal swab sample. Ct: cycle threshold. Gene N (Slope: -4.292, intercept: 51.074, R^2: 0.988 and p-value: $6.3E^{-13}$); Gene Orf1ab (Slope: -2.641, intercept: 38.859, R^2: 0.910, and p-value: $1.041E^{-08}$); Gene S (Slope: -2.934, intercept: 40.289, R^2: 0.956 and p-value: $3.207E^{-10}$).

CC BY

TABLE I

Characteristics and results of the biological samples included in the test of sensitivity and specificity of the RNA-dependent RNA polymerase (RdRp) real-time quantitative polymerase chain reaction (RT-qPCR) assay

ID	Days after symptoms onset	Type of sample	RT-qPCR (in house)	Allplex			Classification
			RdRP gene CT	E gene CT (Allplex)	RdRP gene CT (Allplex)	N gene CT (Allplex)	
176	5	nasopharyngeal swab	29,82	25,99	29,24	29,03	TP
176	5	oropharyngeal swab	Undetermined	33,97	37,74	36,51	FN
179	5	nasopharyngeal swab	20,17	15,12	19,14	18,76	TP
179	5	oropharyngeal swab	25,67	22,12	25,64	25,84	TP
183	4	nasopharyngeal swab	31,93	27,52	30,19	29,85	TP
183	4	oropharyngeal swab	30,56	26,79	31,09	30,6	TP
239	10	nasopharyngeal swab	35,73	31,01	34,73	32,71	TP
239	10	oropharyngeal swab	34,38	29,97	33,44	33,99	TP
285	13	nasopharyngeal swab	36,10	30,12	32,64	33,17	TP
285	13	oropharyngeal swab	32,79	29,25	33,12	33,22	TP
297	7	nasopharyngeal swab	31,38	26,94	30,14	NA	TP
297	7	oropharyngeal swab	31,92	28,02	32,52	NA	TP
300	9	nasopharyngeal swab	31,41	27,31	31,07	30,3	TP
300	9	oropharyngeal swab	35,00	30,86	34,87	34,54	TP
302	10	nasopharyngeal swab	15,99	11,32	13,67	13,39	TP
302	10	oropharyngeal swab	33,18	29,63	32,84	32,19	TP
303	9	nasopharyngeal swab	28,56	23,33	26,54	26,81	TP
303	9	oropharyngeal swab	30,52	26,85	30,53	30,24	TP
304	6	nasopharyngeal swab	21,45	15,05	17,77	17,3	TP
304	6	oropharyngeal swab	27,94	24,17	28,05	27,82	TP
305	6	nasopharyngeal swab	38,02	33,11	34,71	34,51	TP
305	6	oropharyngeal swab	34,96	30,46	33,22	33,29	TP
306	3	nasopharyngeal swab	34,96	30,06	32,78	32,38	TP
306	3	oropharyngeal swab	31,92	27,86	31,6	32	TP
307	9	nasopharyngeal swab	32,43	27,57	30,1	29,86	TP
307	9	oropharyngeal swab	35,17	31,76	34,15	35,23	TP
308	6	nasopharyngeal swab	35,55	29,22	32,34	31,6	TP
308	6	oropharyngeal swab	37,76	33,43	38,28	37,52	TP
309	8	nasopharyngeal swab	23,70	18,87	20,85	21,47	TP
309	8	oropharyngeal swab	32,32	28,48	32,08	32,46	TP
310	10	nasopharyngeal swab	26,35	21,63	24,06	25,16	TP
310	10	oropharyngeal swab	32,13	27,92	31,24	31,76	TP
311	11	nasopharyngeal swab	36,54	32,69	34,87	35,4	TP
311	11	oropharyngeal swab	25,78	19,17	22,31	21,89	TP
312	11	nasopharyngeal swab	33,62	29,97	33,21	33,21	TP
312	11	oropharyngeal swab	32,24	25,8	29,33	29,01	TP
313	10	nasopharyngeal swab	35,20	31,46	35,36	35,04	TP
313	10	oropharyngeal swab	26,08	20,23	24,04	23,6	TP
314	4	nasopharyngeal swab	Undetermined	NA	NA	NA	TN
314	4	oropharyngeal swab	Undetermined	NA	NA	NA	TN
315	13	nasopharyngeal swab	29,14	24,36	27,04	27,2	TP
315	13	oropharyngeal swab	29,46	25,01	28,08	28,32	TP
316	3	nasopharyngeal swab	28,21	22,71	25,53	25,5	TP
316	3	oropharyngeal swab	27,83	24,23	28,2	28,36	TP
317	8	nasopharyngeal swab	24,10	20,54	23,02	23,46	TP

317	8	oropharyngeal swab	27,41	23,94	27,82	28,6	TP
318	9	nasopharyngeal swab	28,73	22,04	25,24	25,38	TP
318	9	oropharyngeal swab	29,90	25,6	29,18	29,19	TP
319	3	nasopharyngeal swab	17,20	9,28	11,98	12,32	TP
320	17	nasopharyngeal swab	30,49	26,14	28,27	28,4	TP
320	17	oropharyngeal swab	34,14	30,4	33,95	33,18	TP
321	6	nasopharyngeal swab	24,83	19,93	24,1	23,5	TP
321	6	oropharyngeal swab	23,77	19,43	23,1	22,76	TP
322	11	nasopharyngeal swab	31,92	27,35	29,62	28,75	TP
322	11	oropharyngeal swab	33,52	29,6	33,81	33,73	TP
324	18	nasopharyngeal swab	33,96	29,36	32,28	31,83	TP
324	18	oropharyngeal swab	Undetermined	NA	NA	NA	TN
325	8	nasopharyngeal swab	22,15	17,59	20,57	19,81	TP
325	8	oropharyngeal swab	36,29	31,66	35,52	35,64	TP
326	10	nasopharyngeal swab	32,22	26,11	29,74	29,57	TP
326	10	oropharyngeal swab	31,99	28,76	32,11	32,02	TP
327	10	nasopharyngeal swab	33,68	28,94	32,07	31,52	TP
327	10	oropharyngeal swab	Undetermined	NA	NA	39,56	FN
328	13	nasopharyngeal swab	Undetermined	34,26	36,51	35,45	FN
328	13	oropharyngeal swab	Undetermined	34,83	NA	39,02	FN
329	10	nasopharyngeal swab	Undetermined	30,95	36,01	33,75	FN
329	10	oropharyngeal swab	34,45	30,37	33,09	34,05	TP
330	12	nasopharyngeal swab	24,54	19,4	22,96	23,11	TP
330	12	oropharyngeal swab	34,18	30,16	34,18	33,66	TP
331	12	nasopharyngeal swab	36,49	31,77	36,63	35,56	TP
331	12	oropharyngeal swab	32,92	29,03	32,63	32,95	TP
333	11	nasopharyngeal swab	31,19	26,78	29,89	29,35	TP
333	11	oropharyngeal swab	36,29	32,91	36,57	36,07	TP
334	6	nasopharyngeal swab	24,49	18,85	21,34	21,4	TP
334	6	oropharyngeal swab	26,17	22,17	25,39	25,71	TP
335	12	nasopharyngeal swab	23,22	19,56	22,7	22,66	TP
335	12	oropharyngeal swab	27,25	23,98	27,33	27,59	TP
337	13	nasopharyngeal swab	35,70	28,76	33,1	32,03	TP
337	13	oropharyngeal swab	Undetermined	34,55	36,98	38,93	FN
338	7	nasopharyngeal swab	23,98	18,84	22,92	21,49	TP
343	12	oropharyngeal swab	Undetermined	NA	NA	NA	TN
554	1	nasopharyngeal swab	38,82	30,81	37,11	33,04	TP
599	2	nasopharyngeal swab	16,87	11,53	15,05	14,61	TP
721	4	oropharyngeal swab	Undetermined	35,51	NA	37,07	FN
14625	6	nasopharyngeal swab	Undetermined	34,86	NA	35,37	FN
14701	5	oropharyngeal swab	15,74	10,97	15,58	14,14	TP

TABLE II
Number of viral copies per reaction (quantity) and the corresponding cycle threshold (Ct) for each replicate used in the assay

**Quantity**	**Ct**
100	35,20
100	31,30
100	33,57
100	32,24
100	32,41
100	35,76
100	33,86
100	38,58
100	34,65
100	34,28
1000	31,83
1000	30,57
1000	33,01
1000	32,37
1000	32,10
1000	30,93
1000	30,77
1000	33,06
1000	35,59
1000	32,26
10000	28,67
10000	28,36
10000	30,02
10000	29,32
10000	29,27
10000	27,96
10000	27,92
10000	29,48
10000	30,43
10000	28,08
10000	29,56
100000	25,19
100000	25,06
100000	26,65
100000	25,91
100000	26,06
100000	24,81
100000	24,78
100000	25,75
100000	25,96
100000	24,89
100000	26,10
1000000	21,89

1000000	21,93
1000000	23,38
1000000	23,16
1000000	22,76
1000000	21,48
1000000	21,44
1000000	22,27
1000000	22,49
1000000	23,07
1000000	22,86
10000000	18,49
10000000	18,48
10000000	20,02
10000000	19,12
10000000	19,35
10000000	18,23
10000000	18,17
10000000	18,80
10000000	19,03
10000000	19,24
10000000	19,35
100000000	15,19
100000000	15,14
100000000	16,63
100000000	15,58
100000000	16,09
100000000	14,91
100000000	14,29
100000000	15,07
100000000	15,35
100000000	15,73
100000000	16,00
1000000000	12,03
1000000000	11,72
1000000000	13,28
1000000000	11,13
1000000000	12,88
1000000000	11,50
1000000000	11,47
1000000000	11,73
1000000000	11,87
1000000000	12,55
1000000000	12,82
